# Correction: Bacterial recognition by PGRP-SA and downstream signalling by Toll/DIF sustain commensal gut bacteria in *Drosophila*

**DOI:** 10.1371/journal.pgen.1010082

**Published:** 2022-02-23

**Authors:** Shivohum Bahuguna, Magda Atilano, Marcus Glittenberg, Dohun Lee, Srishti Arora, Lihui Wang, Jun Zhou, Siamak Redhai, Michael Boutros, Petros Ligoxygakis

Fig 6 is incorrect. The authors have provided a corrected version here.

**Fig 6:**
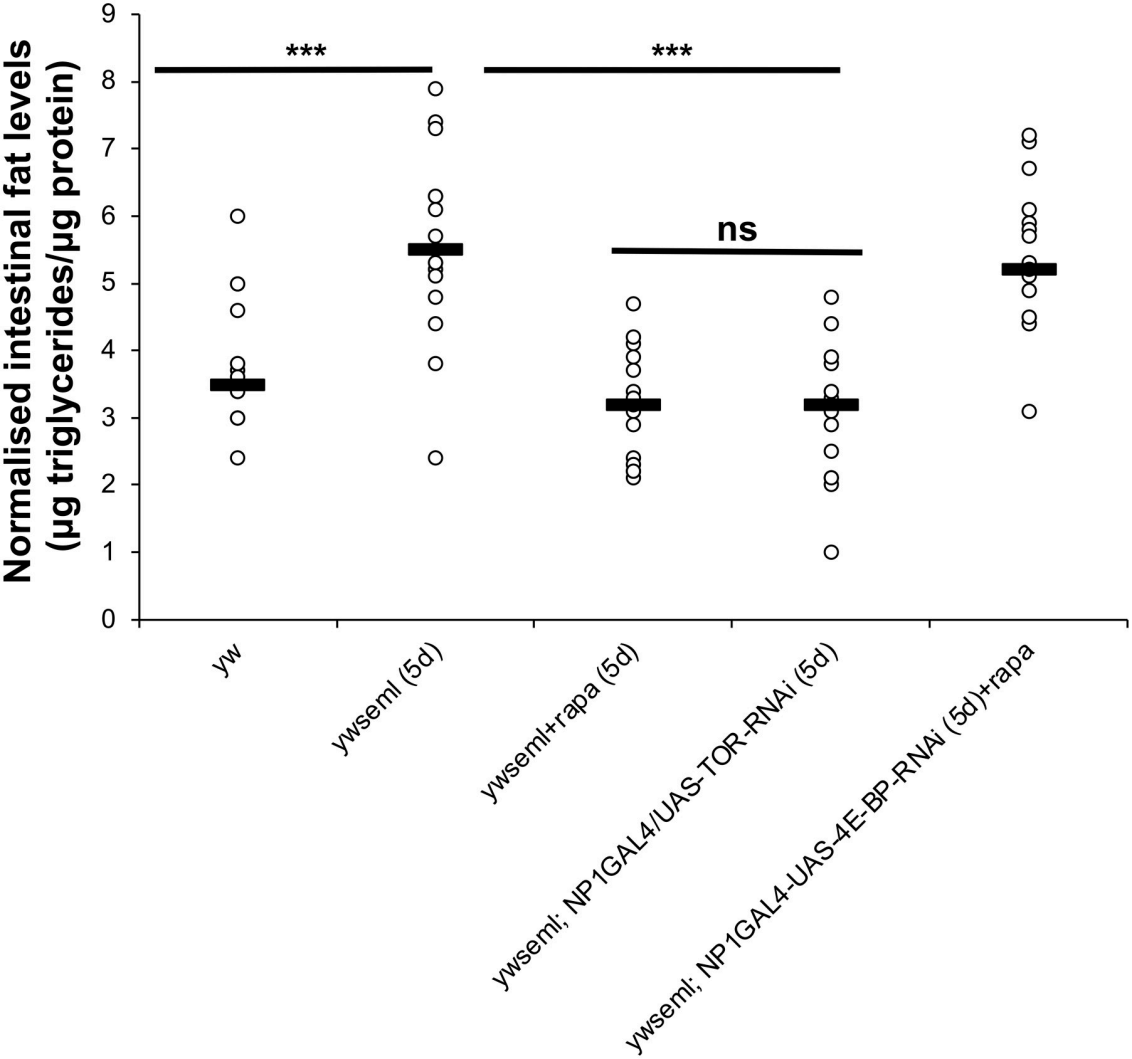
Loss of PGRP-SA increases intestinal fat levels. Loss of PGRP-SA increased intestinal triglyceride levels in 5-day old flies. This phenomenon was suppressed with pharmacological inhibition (rapamycin) or RNAi against TOR in ECs. This was dependent on 4EBP as *yw seml; NP1>4E-BP*^*RNAi*^ treated with rapamycin had fat levels statistically indistinguishable from *yw seml*. N = 15/genotype/treatment a total of three independent experiments (each with n = 5/genotype/treatment). Values of mutants and controls were statistically compared using student’s t-test (***p<0.001, all other comparisons non-significant except *yw seml; NP1>4E-BP*^*RNAi*^ treated with rapamycin compared to *yw*, which has a p value<0.001-comparison not shown in the graph).

## References

[1] Bahuguna S, Atilano M, Glittenberg M, Lee D, Arora S, Wang L, et al. (2022) Bacterial recognition by PGRP-SA and downstream signalling by Toll/DIF sustain commensal gut bacteria in *Drosophila*. PLoS Genet 18(1): e1009992. doi: 10.1371/journal.pgen.1009992 35007276 PMC8782595

