# Supplementary figures and images for: Bacterial recognition by PGRP-SA and downstream signalling by Toll/DIF sustain commensal gut bacteria in Drosophila
Source: PLoS Genet. 2022 Jan 10;18(1):e1009992. doi: 10.1371/journal.pgen.1009992 (PMC8782595; doi:10.1371/journal.pgen.1009992)

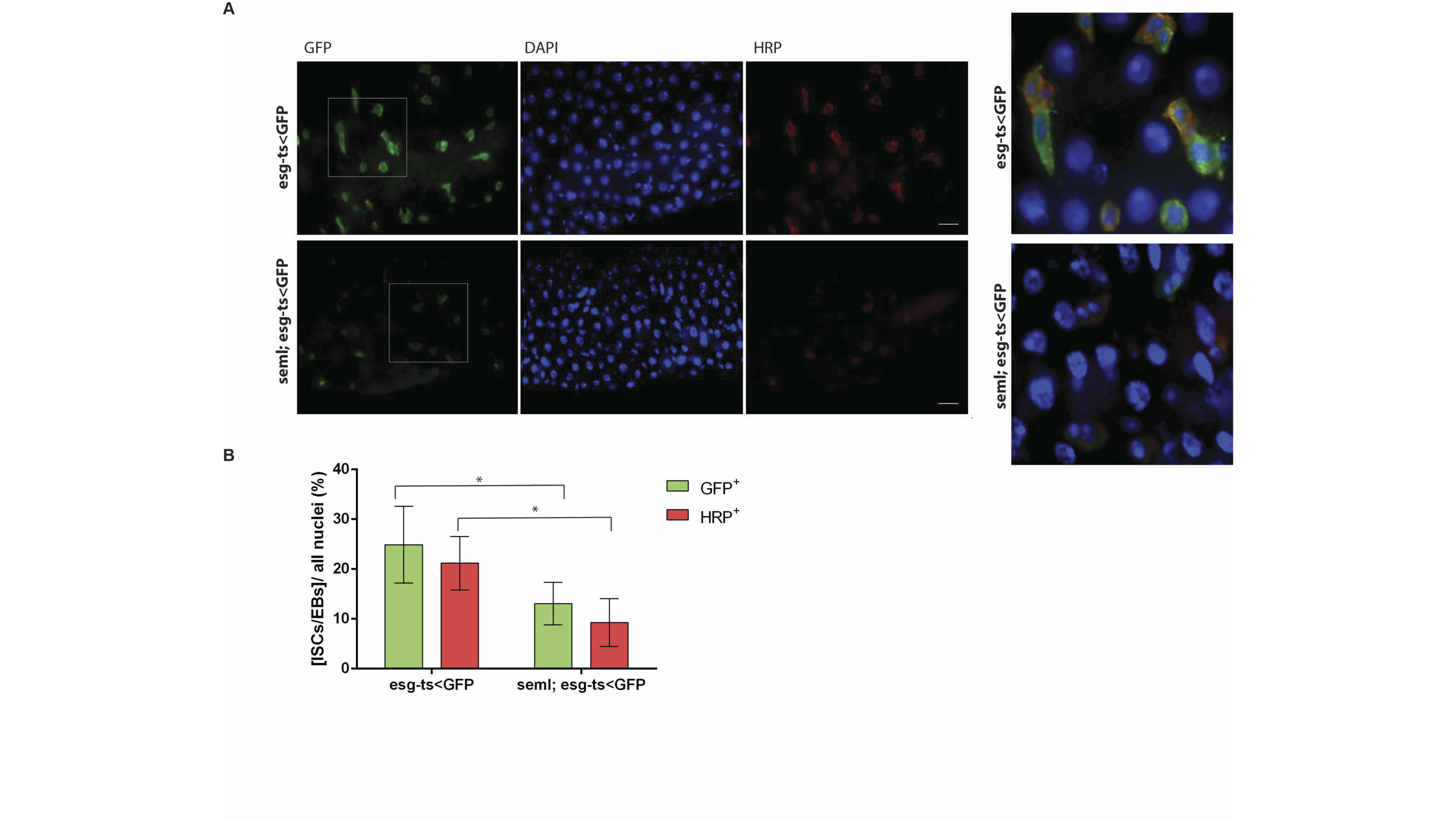

Supplement: S1 Fig — (A) In the absence of infection, ISC (HRP positive, GFP positive) divide to produce EBs (HRP negative, GFP positive). However, in 20-day old flies that were deficient for PGRP-SA this division was not observed (see also insets). (B) Quantification of progenitor cells and ISCs showed that these were significantly reduced (*p<0.05; error bars display 95% confidence intervals, guts from 4 biological repeats were analysed). GFP expression was directed by the UAS dependent mCD8GFP transgene, which marked the cell membranes of the progenitor cells including ISCs and EBs (DAPI all nuclei). (TIFF) [file pgen.1009992.s001.tiff]

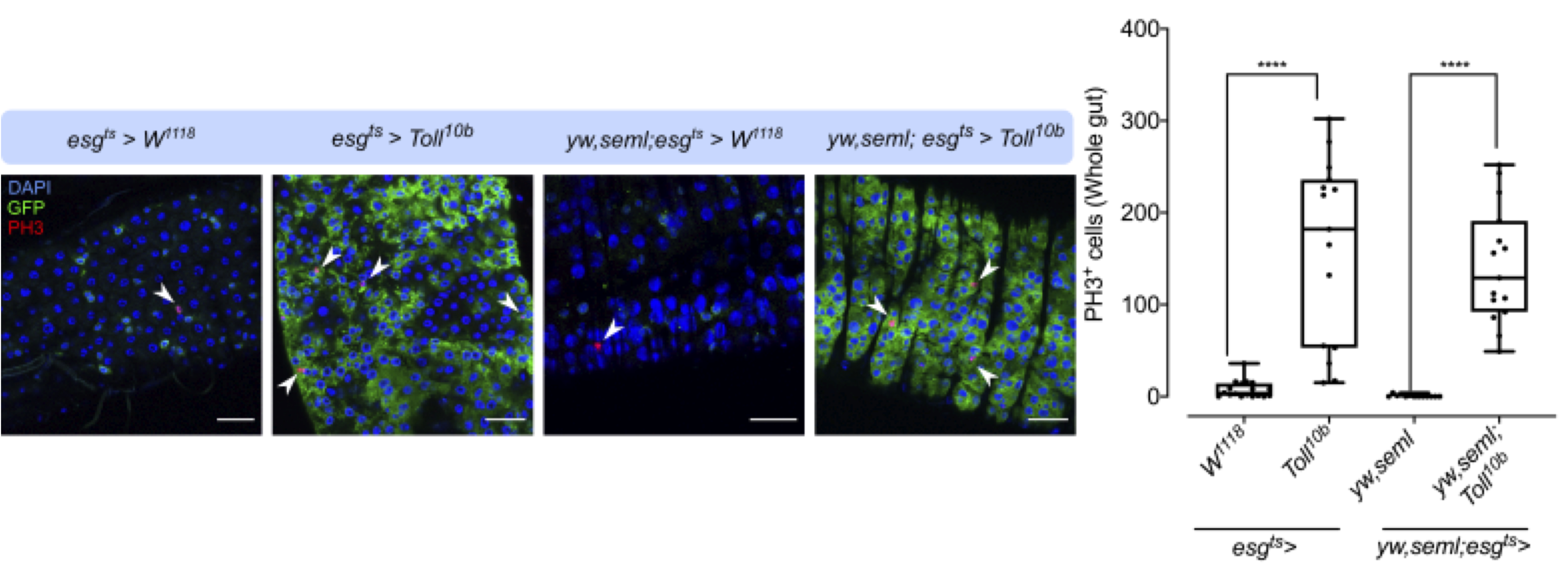

Supplement: S2 Fig — Expressing a UAS- transgene of a gain of function version of the Toll receptor (Toll10B) in progenitor cells activated ISC division in the absence of functional PGRP-SA, as indicated by staining midguts of 5-day old females with an anti-phospho-histone-3 antibody (PH3+) (left panel) and quantified (right panel). Of note, that ISCs are the only PH3+ cells of the intestinal epithelium. (JPG) [file pgen.1009992.s002.jpg]

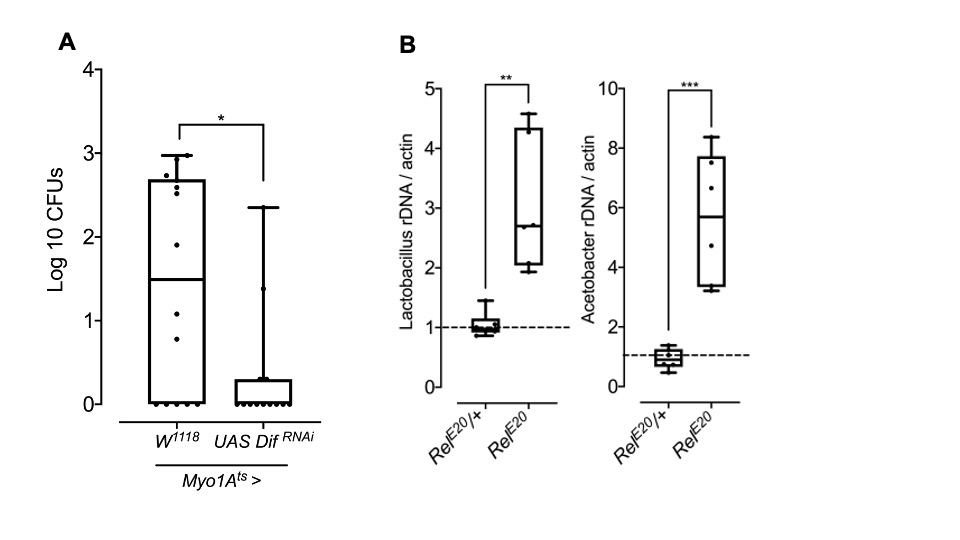

Supplement: S3 Fig — (A) Dif RNAi in enterocytes resulted in a significant reduction in bacterial CFUs. (B) In contrast, loss of Relish resulted in the significant increase of two major components of the intestinal bacteriome namely, Lactobacillus and Acetobacter spp. Statistical comparisons were conducted using student’s t-test (*p<0.1, **p<0.01, ***p<0.001). (JPG) [file pgen.1009992.s003.jpg]

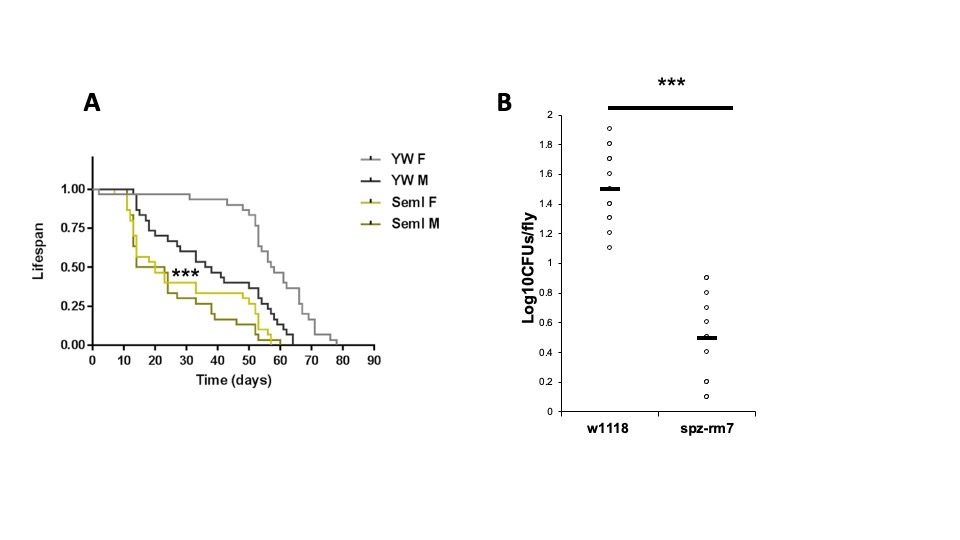

Supplement: S4 Fig — (A) PGRP-SAseml flies showed a significant reduction in lifespan. Sex-specific pairwise statistical comparisons were conducted using the log-rank test (***p<0.001). (B) spzrm7 flies showed significantly reduced intestinal CFUs. Statistical comparisons were conducted using student’s t-test (ns = not significant, ***p<0.001). (JPG) [file pgen.1009992.s004.jpg]

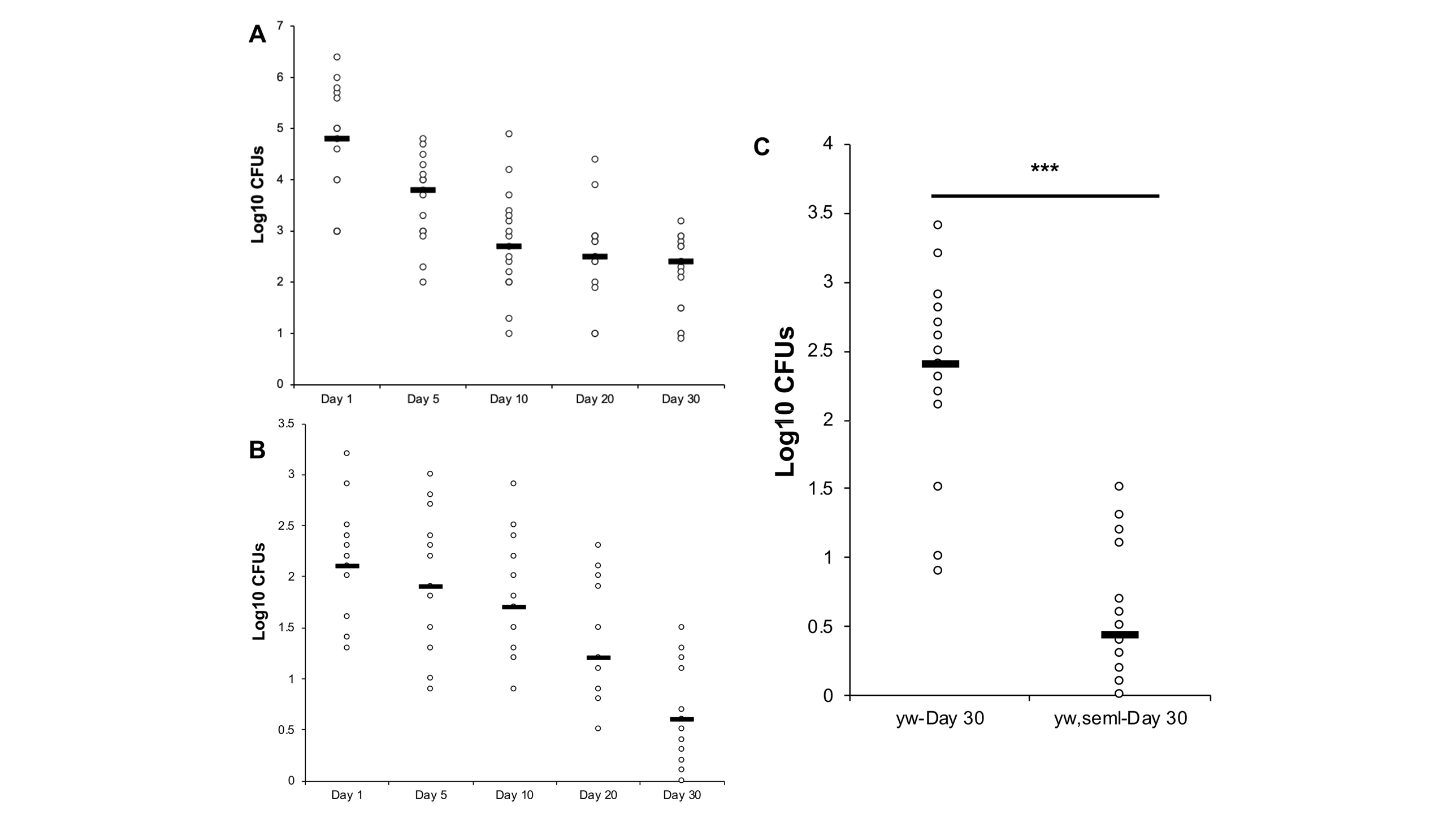

Supplement: S5 Fig — (A) Loss of bacterial density in yw flies was stabilised at day 20 (from day 1 of adulthood) whereas (B) similar treatment of in PGRP-SAseml flies resulted in loss of bacterial density beyond 20-days as (C) was seen in a direct comparison of 30-day old yw and PGRP-SAseml flies. Values of mutants and controls were statistically compared using student’s t-test (***p<0.001). (TIFF) [file pgen.1009992.s005.tiff]

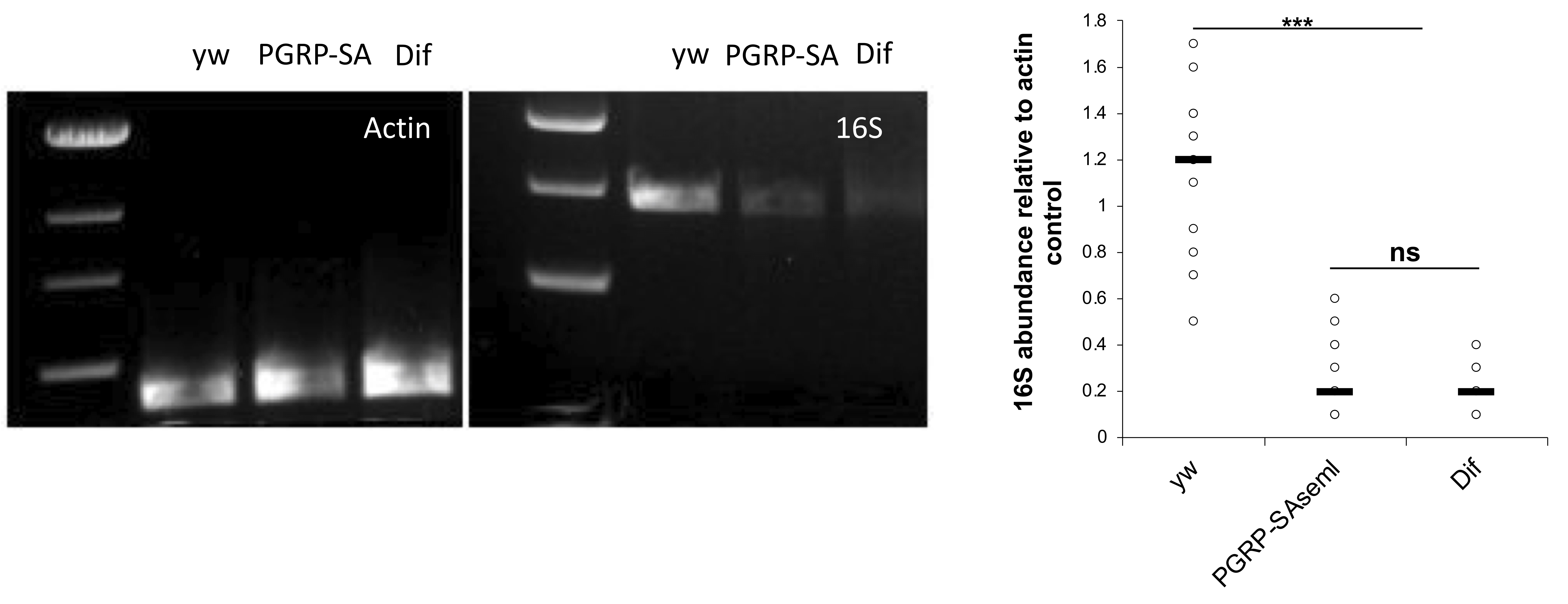

Supplement: S6 Fig — (A) Semi-quantitative PCR of 16S rRNA (right gel) from isolated guts showed an age-dependent reduction in total intestinal bacteria when comparing yw to PGRP-SAseml or dif1 to the internal control (actin; left gel). (B) Quantification of 16S bands (using Image J) relative to actin showed a significant reduction in the quantities of 16S (***p<0.001, ns = non-significant, by student’s t-test). Every dot represents the quantification of one band relative to the respective actin control (n = 10). (JPG) [file pgen.1009992.s006.jpg]

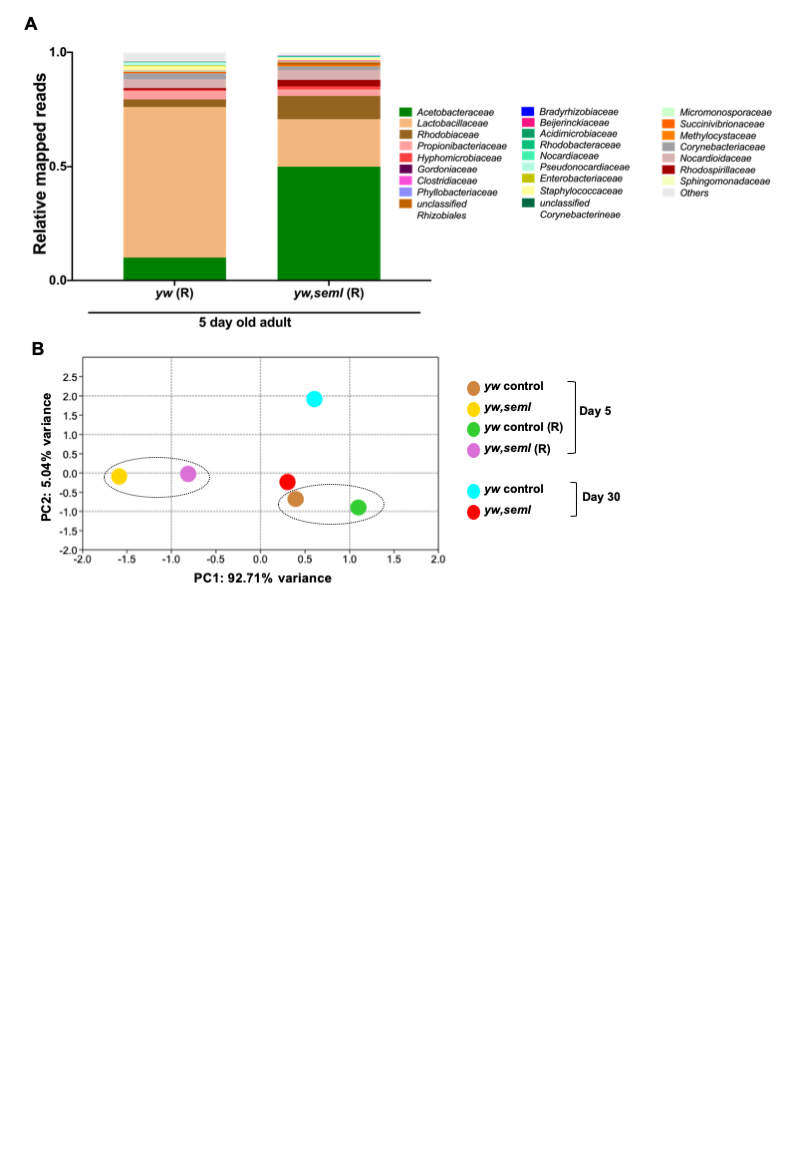

Supplement: S7 Fig — (A) The graph represents the relative abundance of bacterial families observed in the gut of 5-day and 30-day old female yw, ywseml flies revealed by 16S next-generation sequencing. The x axis represents y strains of different ages, and the y axis represents relative mapped reads. (n = 40 guts/strain). (B) PCA plot to show that samples of the same genotype (in circles) were statistically indistinguishable whereas between genotypes were significantly different (***p<0.001). (TIFF) [file pgen.1009992.s007.tiff]

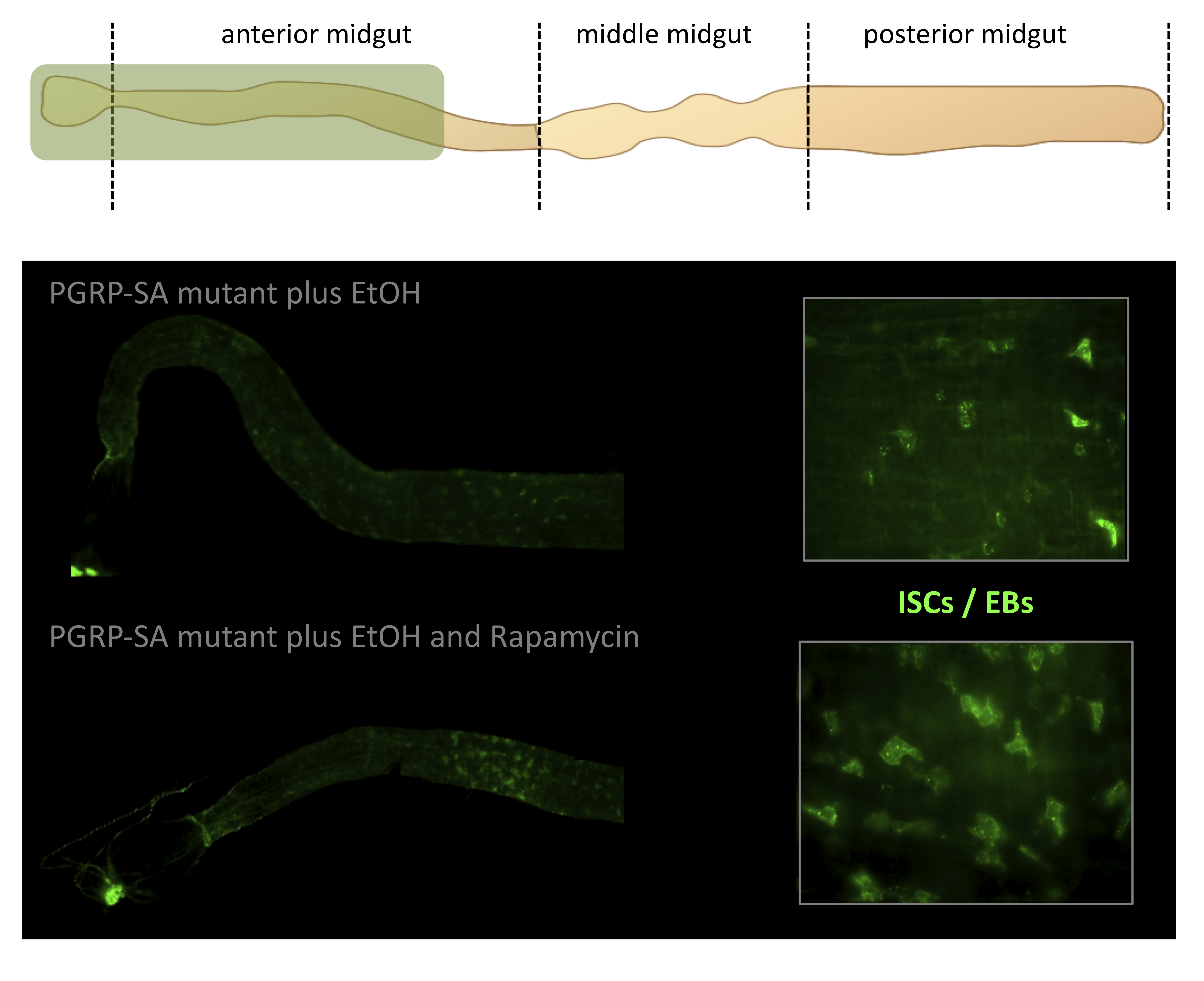

Supplement: S8 Fig — In the anterior midgut (see schematic), IPCs were reduced in PGRP-SAseml mutants. Addition of rapamycin was able to restore them. This is a representative result from 15 guts. (TIFF) [file pgen.1009992.s008.tiff]

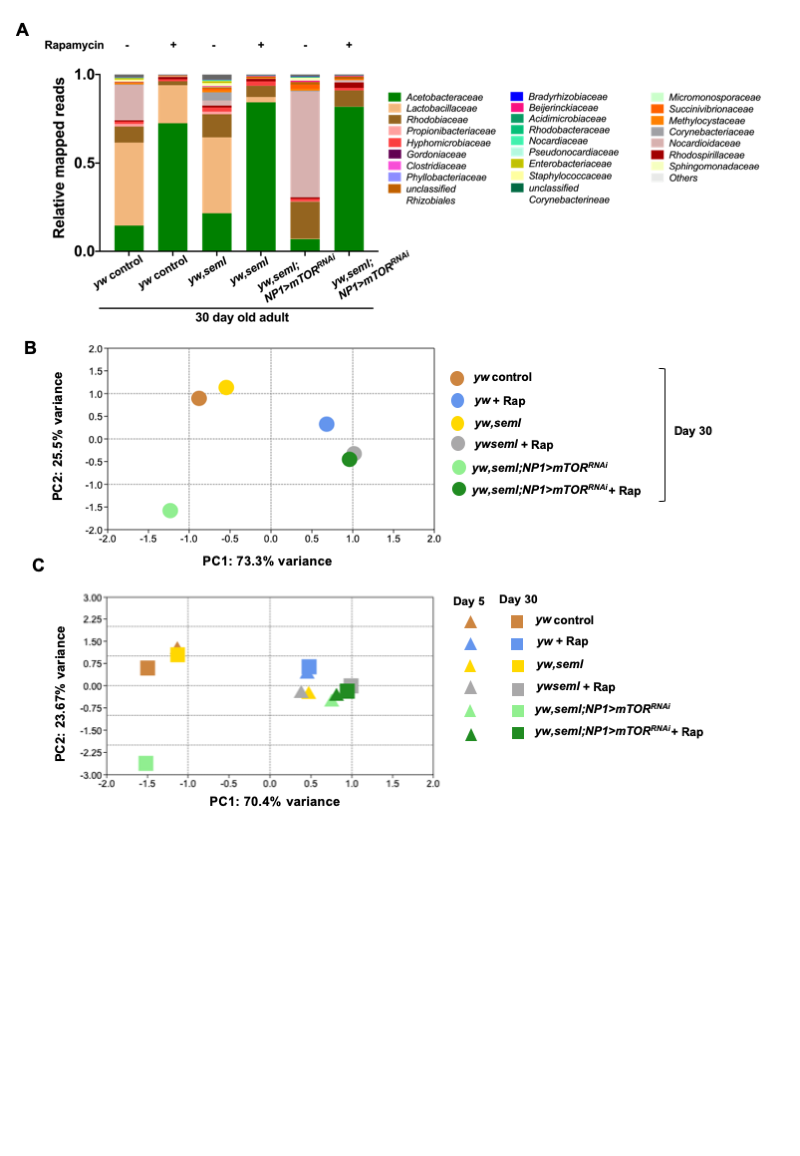

Supplement: S9 Fig — (A). The graph represents the relative abundance of microbial families observed in the gut of 30-day old flies yw, ywseml and flies treated with rapamycin and ywseml; NP1>mTORRNAi (+/- rapamycin) as revealed by 16S next generation sequencing. The x axis represents y strains of different ages, and the y axis represents relative mapped reads. (n = 40 guts/strain). (B) Principal component analysis (PCA) of the above 30-day old flies (n = 40 guts/strain). (C) Principal component analysis (PCA) comparing of 5 and 30-day old of the above genotypes (for 5-day old flies see Fig 4). (n = 40 guts/strain). (TIFF) [file pgen.1009992.s009.tiff]

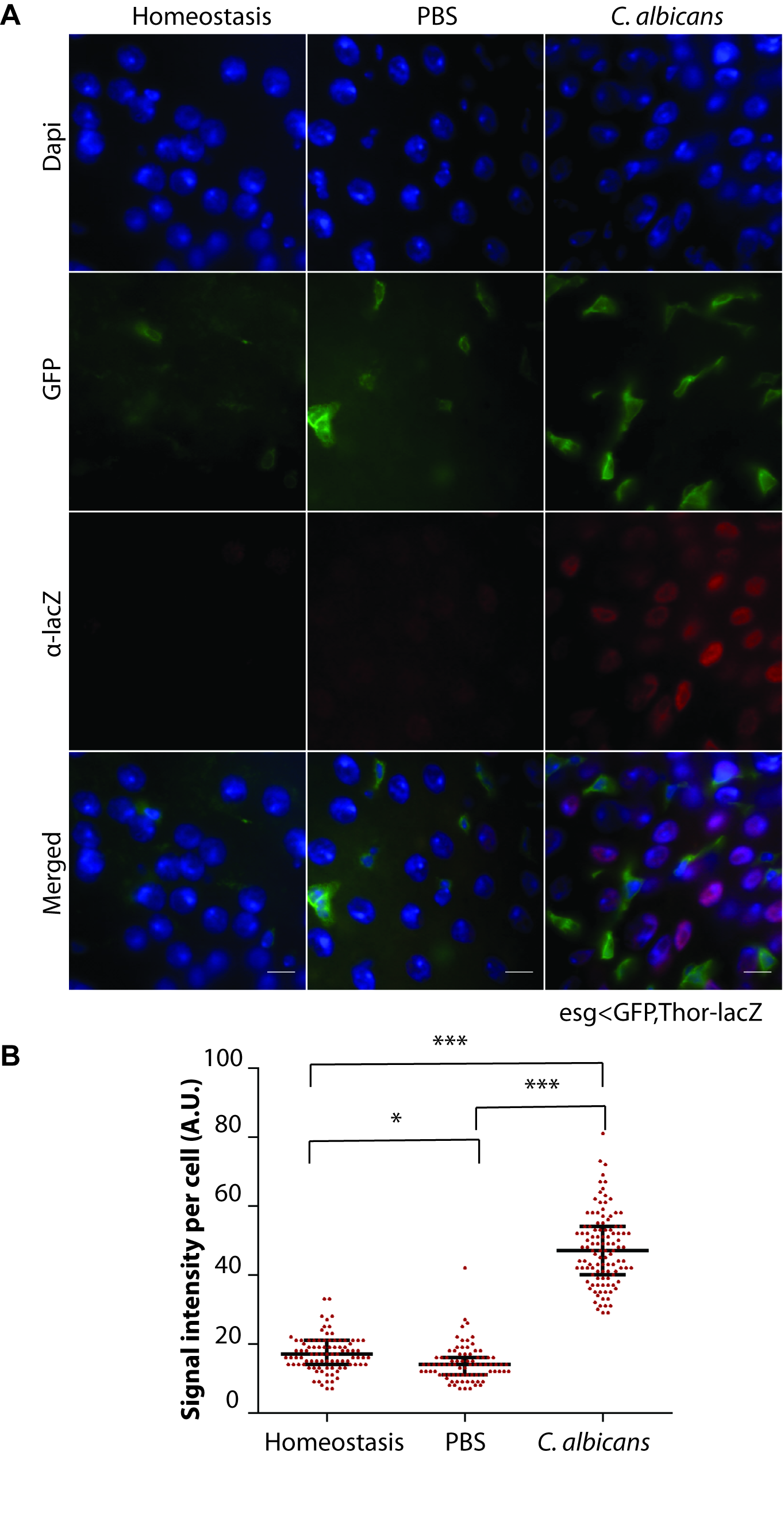

Supplement: S10 Fig — (A) thor-lacZ; esg-ts<GFP flies injected with C. albicans were sampled 36 hours post-infection and compared to non-treated (homeostasis) or those injected with PBS (sterile injury). Gut cells stained with DAPI (blue), anti-β-galactosidase (red) and anti-GFP expressing cells (marking both ISCs and EBs). Shown are representative images from the anterior midgut taken at 63x. (B). Quantification of thor-lacZ expression upon systemic infection. Intensity measured using ImageJ, subtraction of the background was performed for all samples. Ten guts were analysed (approximately 50 cells analysed per gut), 95% confidence intervals displayed, *p<0.05, *** p<0.001. (TIF) [file pgen.1009992.s010.tif]

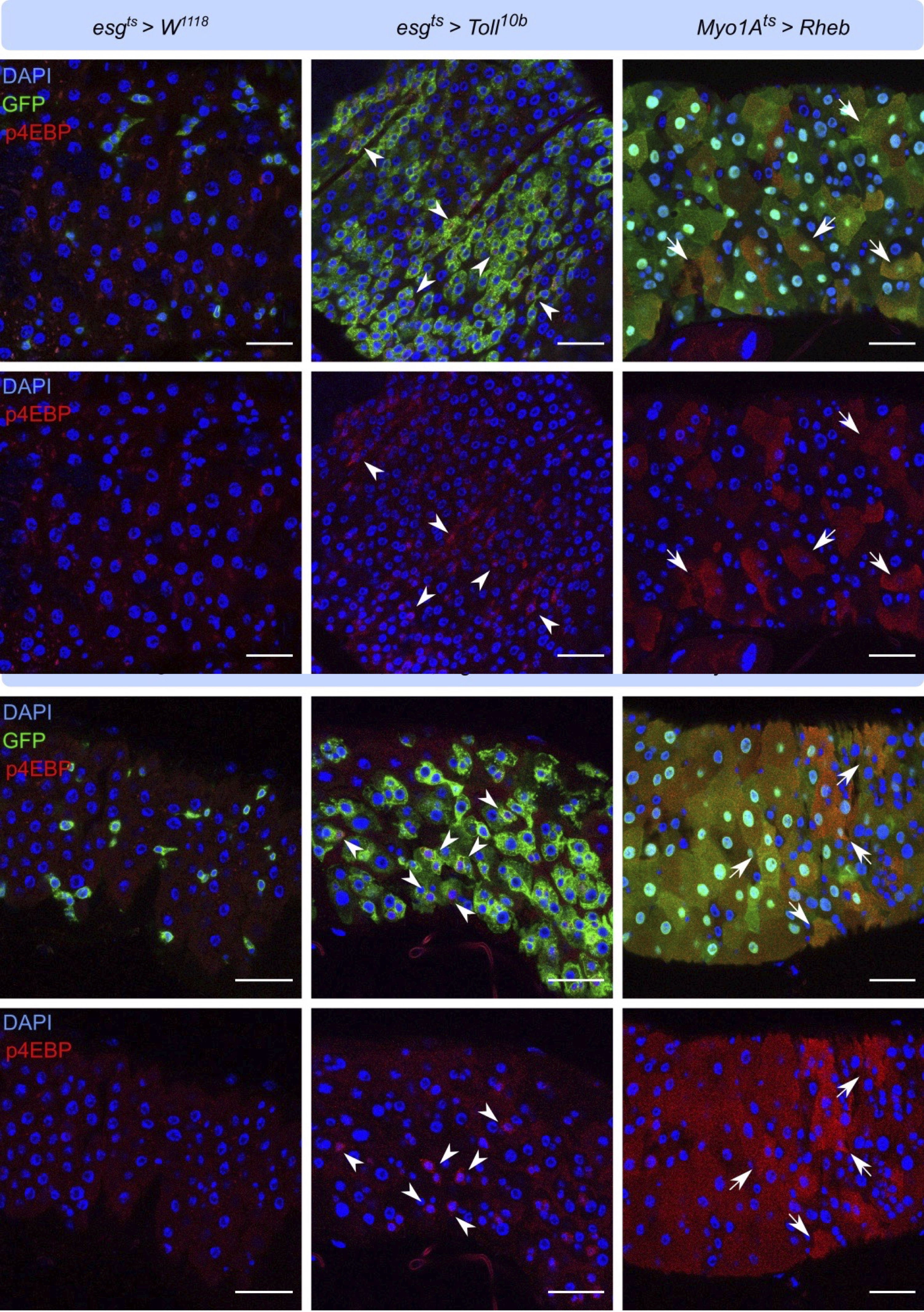

Supplement: S11 Fig — Over-expression of the constitutive form of the Toll receptor, Toll10B, in progenitor cells (GFP-labelled in Toll10B panels) caused a cell-autonomous increase in the occurrence of the phosphorylated form of 4E-BP (p4EBP, arrows) even in the absence of functional PGRP-SA (lower panel). Myo1Ats>UAS-Rheb was used as a positive control for p4EBP staining in enterocytes (GFP labelled in UAS-Rheb panels). Bar is 30 μm. (JPG) [file pgen.1009992.s011.jpg]

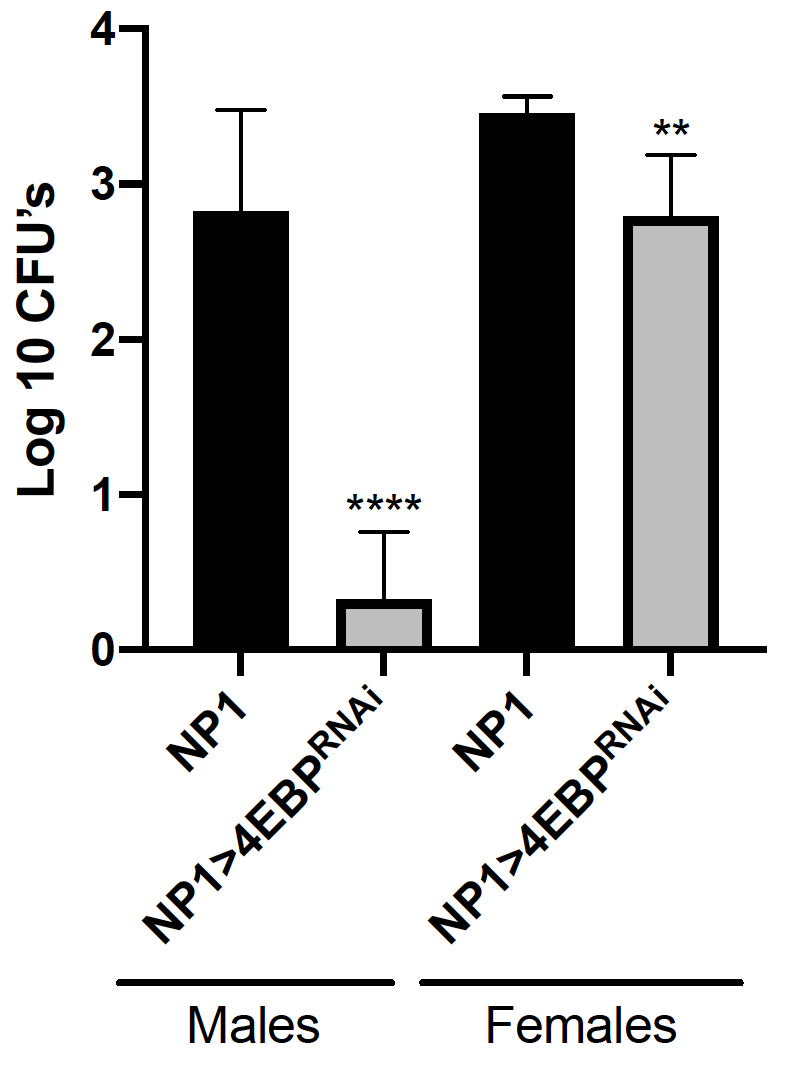

Supplement: S12 Fig — Depletion of 4EBP via RNAi in enterocytes, reduced CFUs in both males and females when compared to the NP1-GAL4 driver (****p<0.0001, **p<0.01 student’s t-test). (TIFF) [file pgen.1009992.s012.tiff]

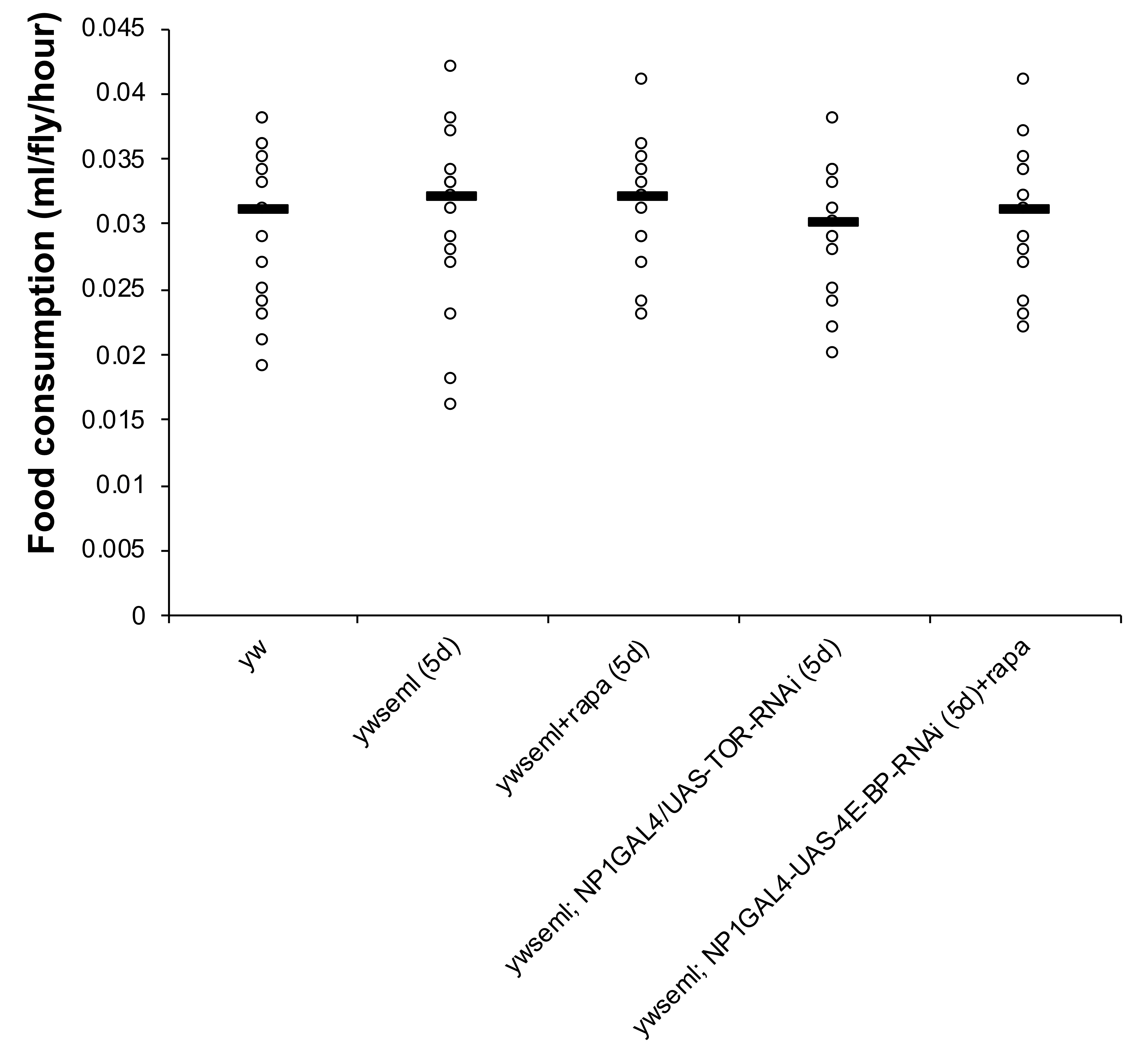

Supplement: S13 Fig — Food consumption was measured by the CAFE method in mated females from day 1 to day 5 of adulthood; each dot represents a vial (of 10 flies each) (n = 15 vials for each genotype and treatment); no comparison was statistically significant (p>0.05, unpaired t-test). (TIFF) [file pgen.1009992.s013.tiff]
