# Supplementary material for: Bacterial recognition by PGRP-SA and downstream signalling by Toll/DIF sustain commensal gut bacteria in Drosophila
Source: PLoS Genet. 2022 Jan 10;18(1):e1009992. doi: 10.1371/journal.pgen.1009992 (PMC8782595; doi:10.1371/journal.pgen.1009992)
Supplement: Attachment — Submitted filename: RESPONSE TO REVIEWERS BAHUGUNA et al.pdf [file pgen.1009992.s014.pdf]

RESPONSE TO REVIEWERS BAHUGUNA *et al*, 2021
PGENETICS-D-21-00436

Reviewer #1: This manuscript from Bahuguna and co-workers is an interesting exploration of the role of the Toll pathway in the interaction between Drosophila and its gut microbiota. The topic is of interest and the work appears sound, but there are a few points where the manuscript and/or data are unclear and a few experimental points that will require attention.

1. *The data presented are consistent with the (very interesting) model at the end. However, there are some experiments that should be done to firm up this conclusion. In particular, the model postulates that the normal role of the Toll pathway in this context is, ultimately, to enable brummer-mediated triglyceride catabolism in the intestine. If this is the case, uncomplicated NP1-driven brummer knockdown should phenocopy the data shown for seml and dif with regard to intestinal bacteria. If this were shown, it would lend credence to the model as a whole.*

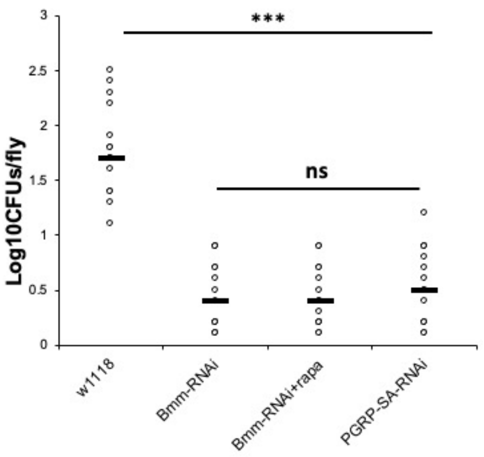

RESPONSE: We thank the reviewer for this observation, which should indeed be the logical conclusion of our model. This is having now been verified (with knocking down the *bmm* gene in enterocytes through the *NP1-GAL4* driver) and presented in the new Fig 8A. We found that *bmm*-RNAi in enterocytes resulted in significantly reduced enteric CFUs statistically indistinguishable to *PGRP-SA-RNAi* in the same cells (see Figure opposite).

2. *The triglyceride measurements are unclear. Do they show whole-fly triglyceride (as implied by the graph in the figure) or intestinal triglyceride (as implied by the text)? In this context, it's important to show intestinal triglyceride specifically. Given the sometimes-ambiguous interpretation of commercial triglyceride assays like that used here, the authors should verify this reading by staining triglyceride in dissected intestines with a neutral lipid stain (Oil Red O, for example, is inexpensive and works well).*

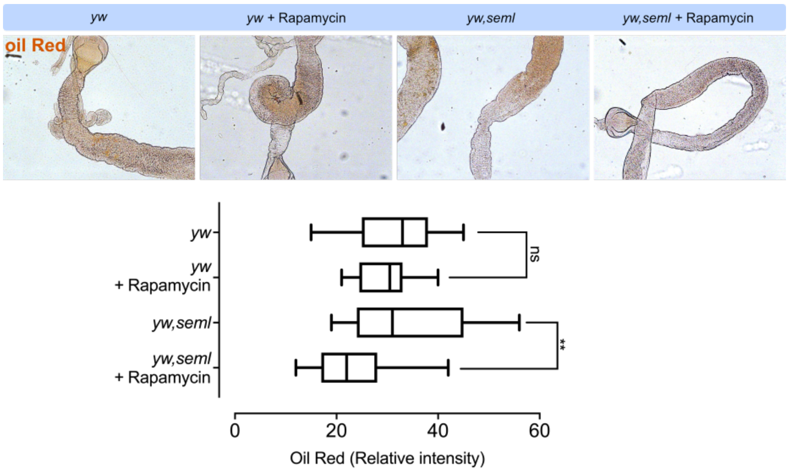

RESPONSE: Apologies for the misunderstanding and mislabelling of the figure. These are gut-only measurements. As shown, we were able to ascertain that female *PGRP-SA*^{seml} flies treated with rapamycin had less oil red staining than untreated *PGRP-SA*^{seml} guts when flies (see figure above). The difference between *yw* and *yw*

+ Rapamycin was at the limit of significance (p=0.15). We have also introduced Oil red staining and their quantification throughout (see new Figures 8 and 9).

3. *It looks as though all experiments were done in females, except the triglyceride measurement, which was done in males. Please show the female data throughout—there are big differences between the sexes in intestinal physiology and switching back and forth between them can be misleading.*
RESPONSE: This has now been rectified.

4. *Some of the experiments are missing controls. There are no positive controls (wild type) shown in Figure 3A or Figure 5. Wild-type samples done in parallel should be shown.*
RESPONSE: In Figure 5A and 5B, the wildtype control is equal to 1 (dashed line). For the others namely Fig 3 and panel 5C this was rectified and made clearer in the legend.

5. *The quantification in Figure S3 appears to be a single experiment. Where did the significance measurements come from?*
RESPONSE: S3A is indeed depicting one representative gel from one experiment while S3B is the quantification for 3 such gels. The panel in S3B has been now replaced by a scatterplot. This figure is now the new Figure S5.

6. The introduction is really absurdly long, but it doesn't cover the really relevant literature on Vibrio cholerae use of Drosophila intestinal triglyceride (Kamareddine et al. Nature Microbiology 3: 243–252 and Liimatta et al. Applied and Environmental Microbiology 84: e01113-18).
RESPONSE: We have shortened the introduction and added the Hang *et al*. Cell Host Microbe 16:592–604 (ref 49 and lines 451-452), which preceded Liimatta *et al.* when discussing how to prove that commensal bacteria directly use lipids.

Minor points

7. The graph labels in Figure 5C must be wrong?
RESPONSE: This has been rectified.

8. The methods don't include details of the genetic introgression of the dif and seml mutants onto the DGRP line.
RESPONSE: This has been rectified (see lines 475-483).

Reviewer #2: The manuscript entitled "Bacterial recognition and Toll signalling sustain commensal gut bacteria in Drosophila" describes the identification of the role of PGRP-SA receptor protein in the maintenance of commensal bacteria in drosophila gut through control of lipid metabolism. Interaction between commensal bacteria and the host physiology and especially immune system is the focus of intense research in the drosophila and mammals model systems. Several papers addressed the action of gut microbiota on gut morphology and the modification of gut metabolism in response to infection. In the present manuscript,

the authors address the question of the maintenance of gut microbiota through interaction with the gut and propose that bacteria are sensed by PGRP-SA which allows, via control of TOR and 4EF-BP, allocation of lipids to the gut lumen favorizing the maintenance of commensal gut bacteria. However, this model is far from being demonstrated by the presented work.

The main concern is related to the article of Vandehoef et al published in Cell Reports 2020. This article clearly shows that the NF-kB/Relish host signalling function in midgut enterocytes is vital to adapt gut microbiota species abundance and composition to host diet macronutrient composition through the control of 4E-BP/Thor and cap-dependent translation. This reference is only cited in the introduction (line 99) without any mention of Relish. Given the extensive similarity on part of the data between this paper and the present submitted manuscript (control of gut microbiota by a NFkB factor through the activity of 4E-BP), this should absolutely be discussed in the present manuscript. Furthermore, the authors make no reference to Molaei et al published in Developmental Cell 2019 that links Relish and Bmm for the control of lipolysis in the fat body. One important point raised by the articles is the involvement of Relish, the transcription factor of the IMD pathway in the control of gut microbiota. Relish and the IMD pathway are known to be active in the gut, activated by commensal bacteria, which has not been shown for PGRP-SA and the Toll pathway. As a consequence, everything that is described in the present manuscript could be due to Relish activation and this should absolutely be tested. Given that the involvement of the Toll pathway is not demonstrated (see below) and that there are questions about the expression of PGRP-SA in the gut, this manuscript can't be accepted without clarification of this major point: the possibility that PGRP-SA activates Relish in the gut, the verification that PGRP-SA is really expressed in the gut. If Relish is indeed activated downstream of PGRP-SA, then the whole part concerning 4E-BP and Bmm is already published in Vandehoef et al. The focus of this work would be to understand how PGRP-SA sense bacteria and activate Relish.

RESPONSE: In their study (Fig 1A), Vandehof *et al* 2020, they show that knocking down Relish in the fly gut does NOT result in a reduction of intestinal CFUs (something that is the case of *Dif, spz* and *seml* mutants, which all belong to the TOLL pathway).

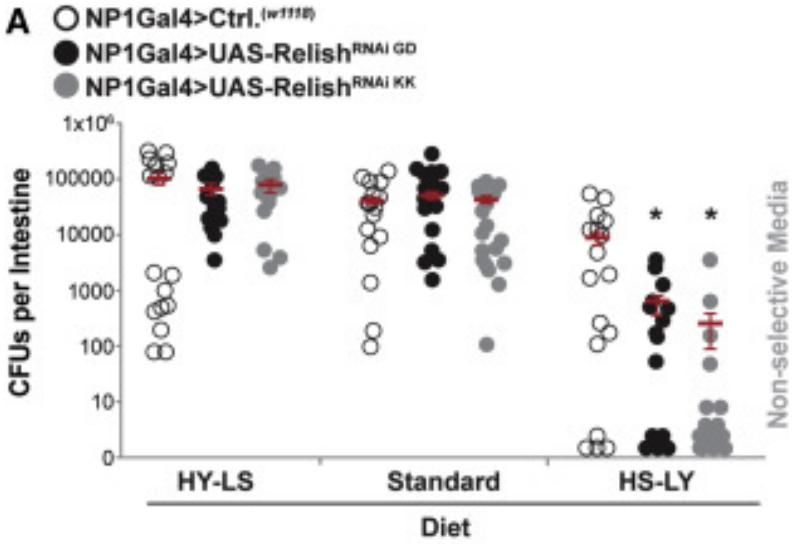

Moreover, *Lactobacillus* and *Acetobacter* 16s rDNA levels, significantly increase in *Relish* mutants compared to *Relish/+.* The latter is statistically indistinguishable to the wildtype w^{1118} control (dashed line, in figure opposite). This is a new S3. In contrast, RNAi of *Dif* in enterocytes significantly decreases enteric CFUs (see figure below).

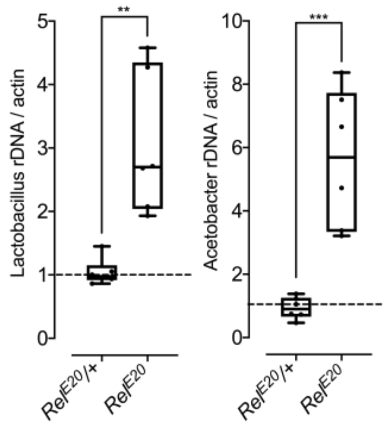

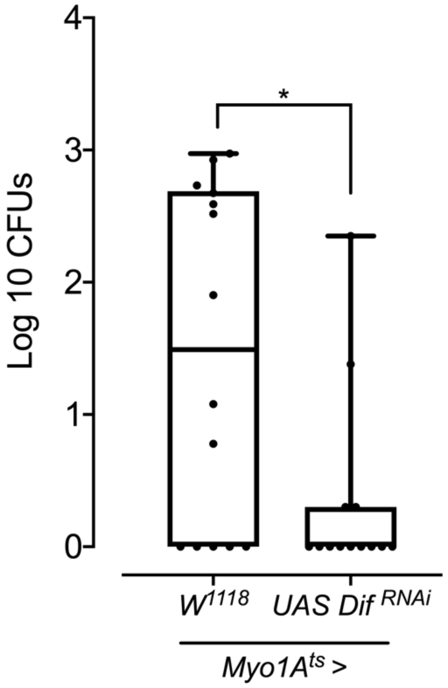

Before going to specific points, I would like to say that the logic of the text is quite difficult to follow. It would need a substantial work in order to clarify either the rational for some experiments or the conclusions raised from data. Furthermore, the manuscript contains a substantial number of mistakes that could have easily corrected before submission.

The title is clearly not supported by the data:
There is no demonstration that bacteria are sensed, except by the fact that PGRP-SA is a circulating receptor shown to activate the Toll pathway. But PGRP-SA was described to be secreted in the hemolymph and we don't know is PGRP-SA could be found in the gut lumen. In the various data accessible on Flybase, PGRP-SA has not been shown to be expressed in the gut. There is one experiment showing that its inactivation specifically in the gut gives the same phenotype as inactivation in the whole flies. To clearly say that bacteria are sensed by PGRP-SA, it requires to demonstrate that PGRP-SA is indeed expressed in the gut.

RESPONSE: In their 2013 paper, *Marianes* and *Spradling AC* (eLife 2: e00886) show that PGRP-SA is regionally expressed in the anterior midgut.

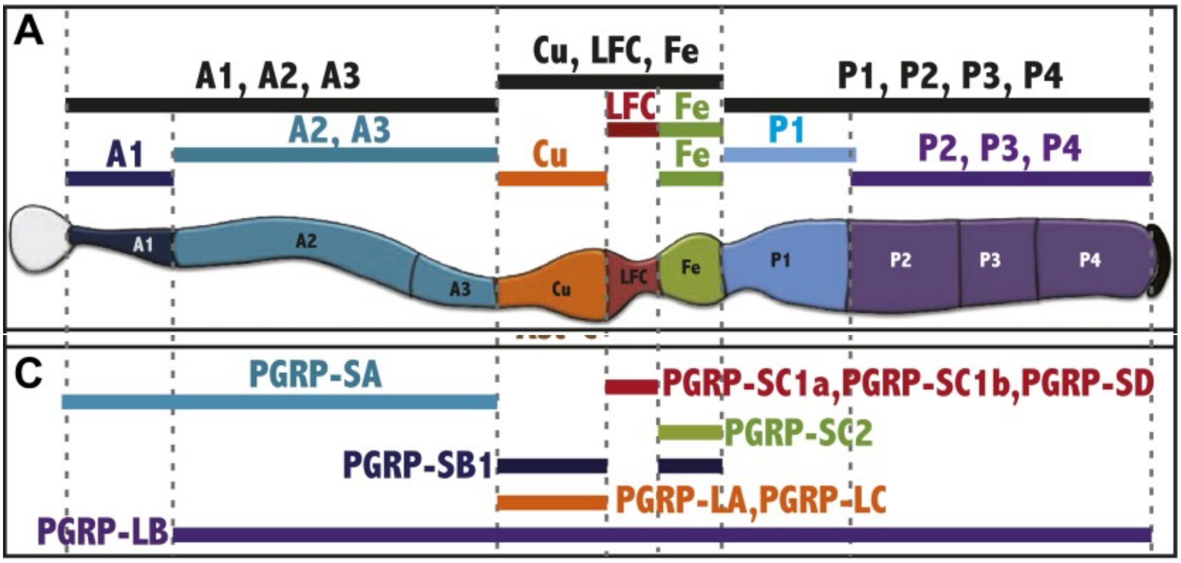

Moreover, according to data released by the Fly Cell Atlas project (Li *et al,* 2021 bioRxiv see https://doi.org/10.1101/2021.07.04.451050), PGRP-SA localizes to a certain degree in the anterior midgut EC cluster (see below).

PGRP-SA

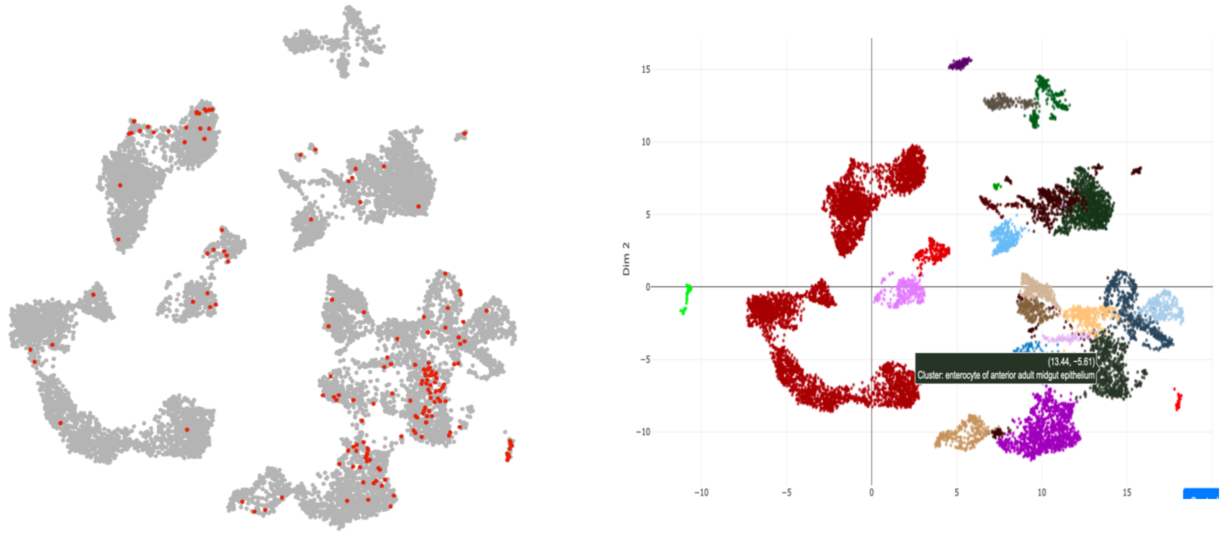

If bacteria sensing is involved, axenic flies should have an accumulation of intestinal fat. Is it the case? The demonstration that PGRP-SA could indeed bind to some of the commensal bacteria would be nice but probably a bit difficult.

RESPONSE: We thank the reviewer for this idea. We have done two experiments to address this point in addition to showing expression of PGRP-SA in the gut. Firstly, we stained axenic flies with Oil red. Despite the clear upwards trend in the accumulation of lipids in those flies

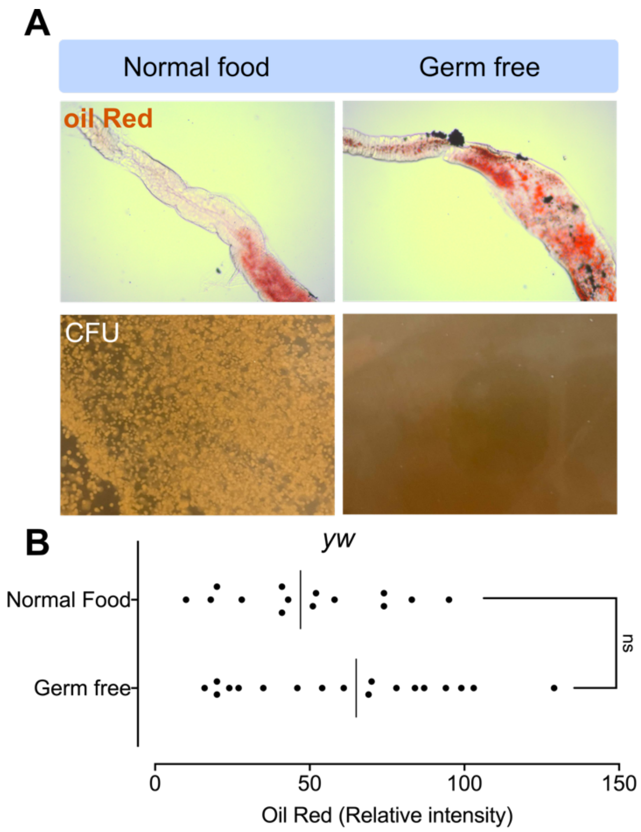

the result was at the margin of statistical significance (p=0.15, see scatterplot opposite). This is the new Fig 9. However, the fact that inhibiting Bmm in ECs significantly increased intestinal lipid accumulation while significantly reducing CFUs indicates the link between the two (new Fig. 8, see response to Reviewer 1). Secondly, we generated three UAS-PGRP-SA point mutation constructs: a) Serine 75 to Alanine (S75A), b) Tyrosine 100 to Alanine (Y100A) and c) Serine 158 to Alanine (S158A). According to peptidoglycan binding studies coupled to the crystal structure of PGRP-SA [Chang *et al,* 2004 PLoS Biol 2(9): e277] S75A enhances binding to the bacterial cell wall since the removal of the hydroxyl group of Ser75 may create a better binding surface for peptidoglycan (PG). In contrast, both S158A and Y100A abolish binding to PG since they are critical for PG binding to the PGRP-SA binding groove (Chang *et al*, 2004). None of the residues chosen for mutagenesis is involved in extensive packing interactions. Hence, alterations of these residues are not expected to disrupt the tertiary structure of PGRP-SA (Chang *et al*, 2004). We then expressed each of these UAS-constructs in enterocytes (through NP1-GAL4) to rescue the *PGRP-SA^{seml}* phenotype of bacterial density loss (measured in 5-day old flies). As expected, expressing a full-length wild type UAS-PGRP-SA transgene was able to restore normal bacterial CFUs as was the S75A mutant UAS--*PGRP-SA^{S75A}*. However, the *UAS-PGRP-SA^{S158A}* or the *UAS-PGRP-SA^{Y100A}* transgenes were unable to rescue bacterial density indicating that the ability of the protein for binding to peptidoglycan needed to be intact. This is the new Fig. 10.

Toll signalling is not demonstrated: the loss of PGRP-SA could result in the loss of Toll signalling, but it is not proved. There are no data showing that Toll signalling is activated in the gut. The authors mentioned drs-GFP flies in the method, but they have not been used. Is there an expression in the gut? Depending on bacterial load? Is it diminished in axenic flies? The only other component of the pathway tested is DIF and only in figure 1 for its effect on bacterial load and its inactivation was not specifically in the gut. The authors also show that the life span of dif mutants is shorten but surprisingly it is the only experiment in which they don't test PGRP-SA mutants.

RESPONSE: We have done two experiments here:

1. Compared to their *yw* genetic background *PGRP-SA^{seml}* flies have a significantly reduced lifespan (new Fig S1B).
2. We have seen that CFUs have been significantly reduced in *spz* flies (new Fig S1C-so along with PGRP-SA and Dif we now have three members of the canonical Toll pathway with the same phenotype).

The authors proposed that fat catabolism down-regulation is the explanation for the lack of commensal bacteria maintenance. The rational for testing intestinal fat level is not explained. Most importantly, I'm not sure whether they really analyzed fat level in the gut or in whole flies since the text and figure legend state that they analyzed intestinal fat level but the figure and the material and methods described whole fly fat level analysis. They should clarify this and test is lipid droplets accumulate in the gut epithelium. The authors proposed that Brummer (bmm) could be implicated in fat catabolism. They should explain in the text the function of Bmm. It's function in the gut was not described yet. Therefore, they should test if its knock-down in the gut impacts intestinal fat level, but also

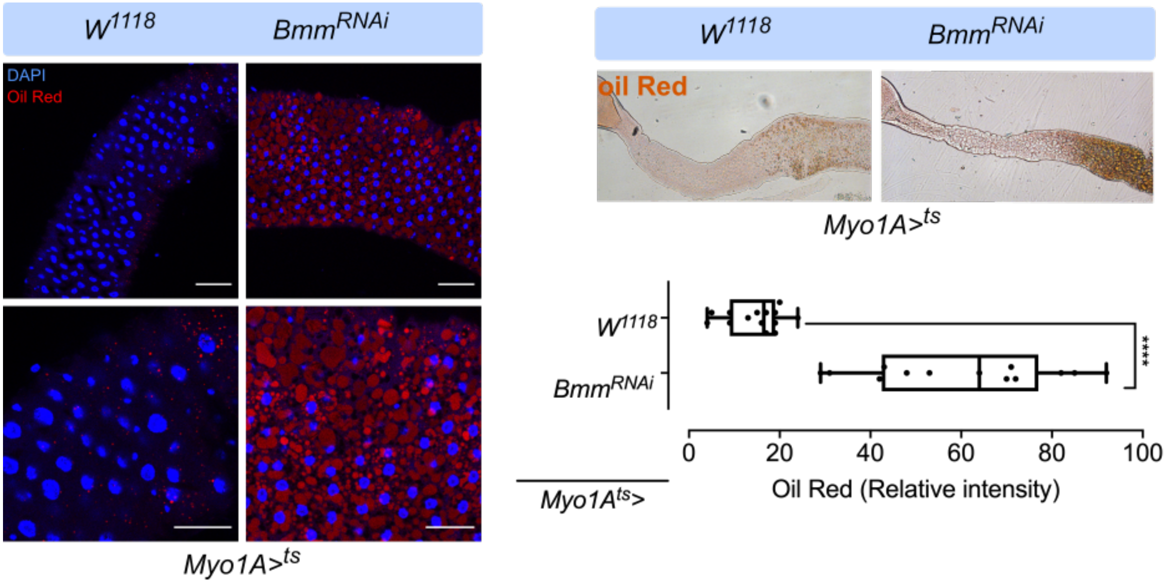

commensal bacteria density. They should also test intestinal fat level in yw seml ; uas bmm RNAI flies treated with rapamycin.

RESPONSE: As mentioned in our response to reviewer 1 we apologise for the conflict between text and figures on this. The measurements were for intestinal triglycerides. Silencing of *Bmm* in enterocytes results in significantly reduced CFUs (see new Fig 8A). Indeed, when *uas-bmm-*^{RNAi} flies were treated with rapamycin the phenotype was not rescued (as it was not rescued when treating *seml;UAS-bmm*^{RNAi} with rapamycin, see Fig 7). In addition, we have shown that RNAi of *Bmm* in enterocytes increases fat levels in the gut (new Fig. 8B).

.

The fact that in 30-day old seml flies, the bacterial density is similar to wild-type levels is quite intriguing. The authors suggest that this is due to bacterial defecation and re-eating. But what makes the gut different that bacteria can live in the gut at 30 days when they were unable at 5 days? Could they verify that these old flies have indeed an increased intestinal fat level to rule out that they adapted their intestinal metabolism? Another point is that in Fig2C they show that in 30-day old yw,seml flies commensal diversity is more similar to 5-day than 30-day old yw flies commensal diversity. Can we suppose that re-eating keeps the commensal in a young state (early colonization of the gut)? The authors should at last comment on that. Putting axenic flies on medium where old flies defected for 24h for example should lead to the same commensal diversity as 30-day old seml flies.

RESPONSE: This is an interesting possibility and we have commented on this in our discussion. However, resequencing the intestinal bacteriome of axenic flies after incubation with medium from older ones to see what happens is beyond the scope of this paper.

Gut microbiota is known to stimulate proliferation of intestinal stem cells. However, figure S1 does not show that there is less proliferation, as stated by the authors (line 209-210) but that there is less ISC and EB progenitors, even if it was published that a small decrease of esg-GFP+ cells is correlated with a reduction of proliferative cells. What is more problematic is that in material and methods, line 662, the authors say that the anterior gut was imaged whereas Broderick et al 2014 showed that it is in the posterior part of the gut that the lack of commensal induces a reduction in esg>GFP staining and proliferation. Is there a consequence of this lack of proliferation on the gut morphology? There is a confusion in the text between intestinal stem cells and progenitors (enteroblasts). They also say (line 208) that, in mammals, Paneth cell are the progenitors or the stem cells of enterocytes. That is not true: stem cells are LGR5+ cells in between Paneth cells.

RESPONSE: We have measured proliferation of Intestinal Stem Cells using a phosphor-histone-3 antibody and there is indeed significantly less PH3+ cells in *PGRP-SA*^{seml} mutants. To avoid confusion, we took measurements for the whole gut (n=15 per genotype). Overactivation of the Toll pathway using a transgene with a gain of function mutation of Toll (Toll10B) in a *PGRP-SA*^{seml} mutant background rescues this under-proliferation and increase PH3+ cells significantly in comparison to the control (see figure below). This is a new experiment presented in the new Figure S2.

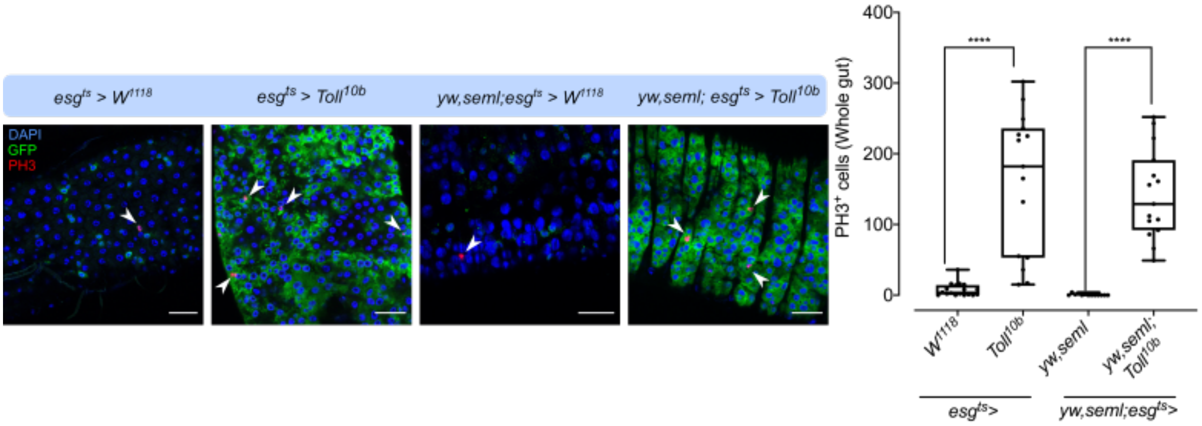

I don't understand the rational for the experiment presented in Fig5B. The authors explain that NF-kB signalling could regulate 4E-BP expression but dTOR regulates 4E-BP activity through its phosphorylation, but the authors are looking at the expression of the gene in PGRP-SA and dTOR knock-down flies. It is possible to test 4E-BP phosphorylation. However, it was shown that Relish can regulate 4E-BP expression. If DIF is really involved in this process, it should be tested that DIF is also able to activate the expression of 4E-BP.

RESPONSE: Toll activation can increase 4E-BP phosphorylation even in a *PGRP*^{seml} background. See below. This is a new experiment and the new Figure S11 (see figure below).

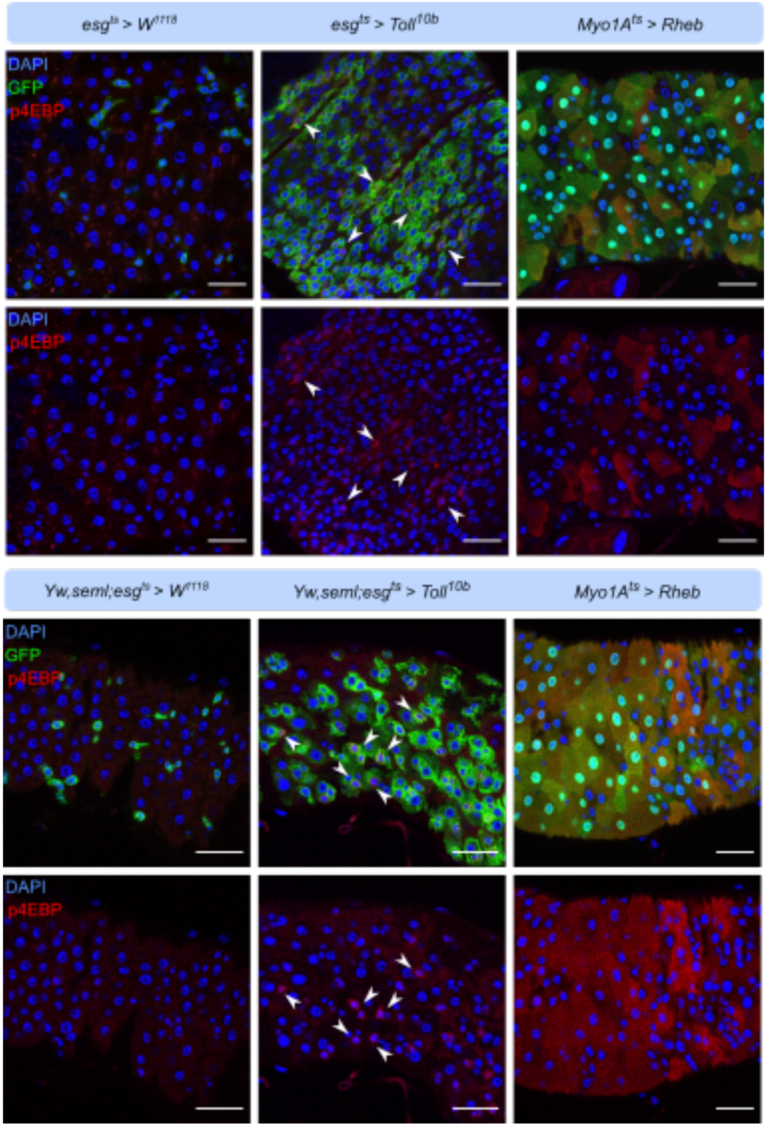

Other more specific questions:

Line 68: please be more precise about the meaning of « host signalling ».

RESPONSE: *This has been rectified.*

Line 320-333: there should be a comment on the fact that rapamycin treated seml flies have a similar bacterial diversity than rapamycin treated flies and therefore that rapamycin treatment is dominant over the seml/wt genetic difference.

RESPONSE: This has been done.

Fig 2B: was the replicate included in this analysis? Figure 2 and S4 could me mixed.

RESPONSE: Figure S4 is a repetition of the experiment shown in Fig 2 after 3 months because there is seasonal variation of the *Drosophila* bacteriome. Nevertheless, there is no statistically significant variation in this context.

Fig S3: how many experiments were done? There is no indication of variation between experiments.

RESPONSE: This has been rectified. The gel in Fig S5A (old Fig S3A) is one representative experiment and Fig S5B (old Fig S3B) is the quantification from 3 such gels.

Fig3A: wt control is missing to see whether the bacterial density is back to normal.

RESPONSE: This has been rectified.

Line 361: be more precise than "regulating gut bacteria". I guess you mean bacterial composition of microbiota.

RESPONSE: We mean bacterial composition of the microbiome, correct. We have changed accordingly.

Line 362: the sentence is unclear: it is 4E-BP coding gene that includes NF-kB binding sites.

RESPONSE: The promoter of *4E-BP* contains NF-kB binding sites.

Line 364: it is unclear what is corroborated by the induction of 4E-BP by C. albicans.
RESPONSE: Activation by the TOLL pathway as Toll is the pathway activated by *C. albicans* infection (see Glittenberg *et al*, 2011 Dis Models Mech). Clarification in lines 334-335.

Figure S5 is missing, replaced by another version of S6.
RESPONSE: This has been rectified.

Fig S7: the name Thor is used for 4E-BP. Since this name is also used in the references cited in the text. The corresponding text uses 4E-BP which is confusing. It would help to use the same name in the whole manuscript.
RESPONSE: *Thor* is the *Drosophila* name for the 4E-BP mutant. We now use 4E-BP throughout.

Fig4A: the authors show the control data to help understand the figure and show variability.
RESPONSE: This has been rectified.

Fig5C: annotation of the figure is wrong (TOR instead of 4E-BP).
RESPONSE: The legend was wrong, and it has now been corrected.

FigS8 and FigS9 have been exchanged.
RESPONSE: This has now been rectified.

FigS9 and line 392: there is no comment on the difference between males and females. In females the reduction of bacterial load is quite low. Is the difference between wild-type males and females significative?
RESPONSE: The difference between wild type males and female is not statistically significant.
